# Microbial inactivation and quality impact assessment of red pepper paste treated by high pressure processing

**DOI:** 10.1016/j.heliyon.2022.e12441

**Published:** 2022-12-20

**Authors:** Henock Woldemichael Woldemariam, Shimelis Admassu Emire, Paulos Getachew Teshome, Stefan Töpfl, Kemal Aganovic

**Affiliations:** a Food Engineering Graduate Program, School of Chemical and Bioengineering, Addis Ababa Institute of Technology, Addis Ababa University, Addis Ababa, Ethiopia; b Center for Food Science and Nutrition, College of Natural and Computational Sciences, Addis Ababa University, Addis Ababa, Ethiopia; c German Institute of Food Technologies (DIL e.V.), Quakenbrueck, Germany; d Osnabrueck University of Applied Sciences, Osnabrueck, Germany; e Department of Food Engineering, College of Biological and Chemical Engineering, Addis Ababa Science and Technology University, Addis Ababa, Ethiopia

**Keywords:** High pressure processing, Inactivation, Microorganisms, Modeling, Optimization, Red pepper paste

## Abstract

The study aimed to investigate inactivation of naturally occurring microorganisms and quality of red pepper paste treated by high pressure processing (HPP). Central composite rotatable design was employed to determine the impacts of pressure (100–600 MPa) and holding time (30–600 s). HPP at 527 MPa for 517 s reduced aerobic mesophilic bacteria count by 4.5 log CFU/g. Yeasts and molds counts were reduced to 1 log CFU/g at 600 MPa for 315 s. Total phenols, carotenoids and antioxidants activity ranged from 0.28 to 0.33 g GAE/100 g, 96.0–98.4 mg βc/100 g and 8.70–8.95 μmol TE/g, respectively. Increase (2.5–6.7%) in these variables was observed with increasing pressure and holding time. Total color difference (*ΔE∗*) values (0.2–2.8) were within the ranges of ‘imperceptible’ to ‘noticeable’. Experimental results were fitted satisfactorily into quadratic model with higher R^2^ values (0.8619–0.9863). Optimization process suggested treatment of red pepper paste at 536 MPa for 125 s for maximum desirability (0.622). Validation experiments confirmed comparable percentage of relative errors. Overall, this technique could be considered as an efficient treatment for the inactivation of microorganisms that naturally occur in red pepper paste with minimal changes in its characteristics.

## Highlights

• Effectiveness of HPP depended on the intensity of pressure and holding time.
• Treatment at 527 MPa for 517 s reduced total plate count by 4.5 log CFU/g.
• Yeasts and molds counts were reduced to 1 log CFU/g at 600 MPa for 315 s.
• Slight increase in total phenols, carotenoid and antioxidant activity was observed.
• Optimization process suggested treatment of red pepper paste at 536 MPa for 125 s.

## Graphical abstract

**Figure:**
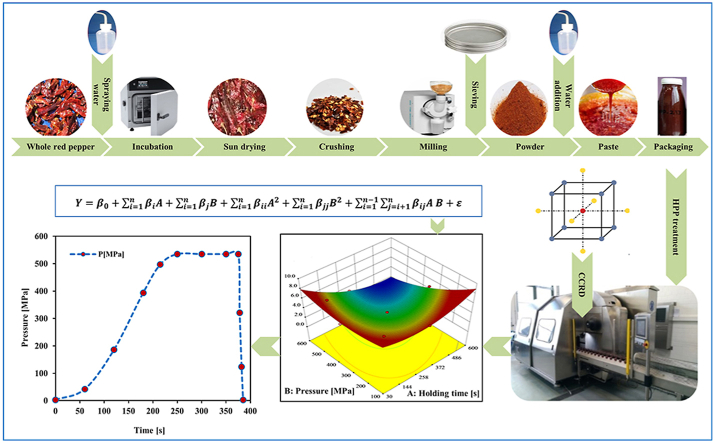

## 1 Introduction

Pepper (*Capsicum annuum* L.), which belongs to the family of *Solanaceae*, is extensively cultivated and consumed globally (Wang et al., 2017) either fresh, dried, fermented or cooked (Pino et al., 2007). Red pepper is highly recognized for its coloring, flavoring and aromatic properties (Dong et al., 2014; Montoya-Ballesteros et al., 2014). In Ethiopia, the powder and its products are widely used as a seasoning agent in the preparation of traditional foods. Red pepper is a rich source of diverse phytochemicals including carotenoids, phenolics, vitamin C, vitamin E, flavonoids, alkaloids, capsaicinoids and quercetins (Bae et al., 2012; Derry et al., 2017; Wang et al., 2017). Due to the potential health benefit, these phytochemicals has gained increased interest for their anti-inflammatory, hypolipidemic, hypoglycemic, analgesic and antioxidative effects (Bley et al., 2012; Victoria-Campos et al., 2015).

Generally, spices are frequently added as raw ingredients in many ready-to-eat foods. They represent effective transmission vehicles of spoilage microorganisms and pathogens that potentially pose public health threat (Dewey-Mattia et al., 2018). Contaminated spices may lead to product recalls and severe foodborne illness (Syamaladevi et al., 2016). It has been noted that red pepper and its products are often contaminated with microorganisms (Jung et al., 2015; Sanatombi and Rajkumari, 2019). As a result, it is imperative to reduce the amount of microorganisms before consumption to as low as possible without affecting the qualities of products (Kyung et al., 2019; Sanatombi and Rajkumari, 2019; Zhang et al., 2020). Several research works have already documented the inactivation of foodborne pathogens in red pepper and pepper-based products by using radio frequency heating (Hu et al., 2018), cold plasma treatment (Mošovská et al., 2018), near-infrared heating combined with ultraviolet light treatments (Cheon et al., 2015) and electron beam treatment (Woldemariam et al., 2021). However, to the best of our knowledge, there are no studies related to inactivation of microorganisms in red pepper paste using novel methods like high pressure processing (HPP) which is recognized as one of the most promising and fastest growing innovative technology.

The mechanism of microbial inactivation with HPP that involves the damage of membrane and cell wall has been reported in previous studies (Simpson and Gilmour, 1997; Briones-Labarca et al., 2011). According to Barba et al. (2012), the impacts of HPP vary with the type of food matrix and intensity of treatment, suggesting each matrix to be investigated independently. Currently, pressure range from 200 to 600 MPa and holding time typically no longer than 5 min at chilled or room temperature are used for the application of HPP for foods (Jabbar et al., 2014; Balasubramaniam et al., 2015; Marszałek et al., 2015; Putnik et al., 2019; Kumari and Farid, 2020). Besides its food preservation potential, several previous works have reported the capacity of HPP to enhance extraction and improve retention of different phytochemicals (Corrales et al., 2008; Patras et al., 2009a; Bimakr et al., 2011).

By increasing treatment conditions, pressure or in certain cases time, the inactivation efficiency is usually enhanced. However, by increasing either of these conditions, the treatment costs increase as well. It is worth of mentioning that the HPP is anyway considered as relatively expensive compared for example to well-established and optimized thermal processing. This points out a need for optimization of processing conditions to achieve desired inactivation level of relevant microorganisms, without wasting energy and overprocessing the food. Thus, it is important to study relationships between pressure and holding time in order to determine optimal processing conditions for required inactivation of microorganisms, while preserving the nutritional and organoleptic qualities of foods (Avsaroglu et al., 2006).

In several previous studies (Kaushik et al., 2016; Fernandes et al., 2017; Irna et al., 2018; Kumari and Farid, 2020), response surface methodology (RSM) was employed efficiently to predict the impact of HPP on the inactivation of microorganisms and various food quality characteristics. However, studies characterizing the efficacy and impact of HPP on red pepper paste have not been getting much attention. Hence, the present study was intended to investigate the inactivation of naturally occurring microorganisms in red pepper paste treated by high pressure processing while maintaining the physicochemical characteristics.

## 2 Materials and methods

### 2.1 Sample collection and preparation

Dried whole red pepper (*C. annuum* L.) sample (10 kg) was procured from a local market inAddis Ababa, Ethiopia and was prepared as outlined in our earlier study (Woldemariam et al., 2021). Briefly, unwanted substances were removed manually and water was sprinkled evenly on the sample in the ratio of 3.5:1 (red pepper/water, m/v) to increase the moisture content. The growth of microorganisms naturally present in the wet sample was facilitated by incubating it at 30 °C for seven days. Then, the stalks were removed from the sample and it was sun-dried and coarsely grounded with cutter mixer (Hobart, USA). The sample was then milled by using laboratory scale hammer mill (Perten Instruments, Finland). Finally, the milled sample was homogenized and packed in polyethylene (PE) bags after being sieved through 0.5-mm mesh. For HPP treatment, distilled water was added to the powder and mixed for 2 min in the ratio of 3:1 (water:powder) using lab-scale mixer (KRUPS 3Mix 700, China) and packed in flexible PET bottles (Nipak BV, Lopik, Netherlands).

### 2.2 Experimental design

A central composite design (CCRD) (Cochran and Cox, 1957) with two independent HPP variables (pressure and holding time) was employed to investigate the response patterns of microbial and physicochemical characteristics of red pepper paste. Holding time was taken as the isobaric holding period without the pressure build-up and decompression at the end of the cycle. For this study, pressure ranging from 100 to 600 MPa and holding time of 30–600 s were used. Further, five-levels of the two variables were coded to lie at ±*α* for axial points, 0 for the center points and ±1 for the factorial points (Table 1).

**Table 1:** Experimental variable ranges with actual and coded levels using CCRD.

Independent variables	Units	Levels of coded variables				
		–*α*	Low	Center	High	+*α*
		–1.414	–1	0	+1	+1.414
Pressure	MPa	100	174	350	527	600
Holding time	s	30	114	315	517	600

The distance between the axial points to the center point was ±1.414, and calculated using α = (2^n^)^1/4^, where n is the number of variables. The codes were calculated as a function of the range of interest of each factor. Based on CCRD, the experimental runs comprised of 10 trials (4 factorial points, 2 center points and 4 axial points). Experiments were performed as a single block and the order of runs within the block was randomized.

### 2.3 High pressure treatment

Ten packed red pepper paste samples (300 mL each) were placed in a pressure basket and loaded horizontally into a pressure chamber of the high pressure system. Pressure treatment was performed using a small industrial scale system (Wave 6000/55 Hiperbaric, Burgos, Spain). At room temperature, the system runs at maximum pressure of 600 MPa and at pressure build-up rate of 100 MPa/min. The high pressure vessel was filled with water by pressure boosting pump, pumping the water from the tank into the chamber, and releasing air on the other side. After the required holding period, the pressure was released and treated samples were unloaded from the pressure vessel. Samples were analyzed for microbiological and physicochemical characteristics immediately after the HPP treatment.

### 2.4 Microbiological analysis

Microbiological analysis was conducted using German official collection of analysis procedures for yeasts, molds (method: ASU 01.00–371991-12A) and aerobic mesophilic bacteria (AMB) count (method: ASU L00.00–88/2 2015-06A). A sample of 10 g was aseptically transferred to a flexible and sterile filter bag to which 90 mL of maximum recovery diluent (Oxoid, Thermo Fisher Scientific, Waltham, MA, USA) was added. The sample and the diluent were homogenized for 1 min with stomacher (Interscience, France). Serial dilutions were made and plated on yeast extract glucose-chloramphenicol-agar (YGC) for yeasts and molds count while plate count (PC) plates (ThermoFisher Scientific, Dreieich, Germany) were used for AMB. Finally, YGC plates were incubated for 4 days at 25 ± 2 °C and PC plates for 72 ± 3 h at 30 ± 1 °C. Colony counts were reported as colony forming units per gram (CFU/g).

### 2.5 Response surface modeling, process optimization and validation

The effects of independent variables on the selected responses have been assessed and the regression models were developed by using RSM. Experimental data obtained were fitted to a second-order (quadratic) polynomial response surface function using Eq. (1):

(1) $$Y=\beta_{0}+\sum_{i=1}^{n}\beta_{i}A+\sum_{i=1}^{n}\beta_{j}B+\sum_{i=1}^{n}\beta_{ii}A^{2}+\sum_{i=1}^{n}\beta_{jj}B^{2}+\sum_{i=1}^{n-1}\sum_{j=i+1}^{n}\beta_{ij}AB$$

where; Y is the response value, A and B are the codded values, n is the number of independent variables, ε is the error term, $\beta_{0}$, $\beta_{i}$ and $\beta_{j}$, $\beta_{ii}$ and $\beta_{jj}$, $\beta_{ij}$ are the constant, linear, quadratic and interaction regression coefficients, respectively.

Process optimization was carried out by employing numerical optimization technique, with desirability ranging from zero to one at the goal. Further, validation experiments were carried out under optimized process conditions and the percentage relative error was calculated using Eq. (2) (Tripathi and Mishra, 2011).

(2) $$Relative\;error\;\left(\%\right)=\left[\frac{Predicted-Actual}{Predicted}\right]\times 100$$

### 2.6 Physicochemical quality analysis of red pepper paste

#### 2.6.1 Determination of total phenols

The content of total phenols of red pepper paste was determined using Folin-Ciocalteu (F–C) colorimetric method. Two grams of sample were mixed with 20 mL of extraction solvent and incubated overnight at 10 °C. The supernatant was taken after centrifugation of 2 mL mixture at 21,380 × g. Folin-Ciocalteu (F–C) reagent (1 mL) was mixed with the extract (0.2 mL). After 6–8 min, 800 μL of aqueous Na_2_CO_3_ (0.7 M) was mixed using vortex. Absorbance was read at 760 nm after 2 h with spectrophotometer (SPECORD 40, Analytik Jena AG, Germany) and total phenols content was expressed as mg of gallic acid equivalents (mg GAE/100 g).

#### 2.6.2 Determination of carotenoids

Carotenoids content of red pepper paste was determined using photometric method. A sample of 1 g was mixed with 20 mL of methanol (99.9% Merck KGaA, Germany) and shaken for 10 min (250 shakes/min) with shaker (SM-30, Edmund Bühler GmbH, Germany). The mixture was subsequently centrifuged at 8965 × g for 10 min at 20 °C and supernatant was collected into 100-mL volumetric flask. The procedure was repeated with 20 mL, 15 mL, 15 mL, 10 mL, 5 mL and made up with methanol up to 100-mL mark. The extract was filtered with 0.45 μm membrane filter. Absorbance was read at 470 nm with spectrophotometer (SPECORD 40, Analytik Jena AG, Germany) and carotenoid contents were expressed as mg β-carotene (mg βc/100 g).

#### 2.6.3 Determination of antioxidants activity

The antioxidants activity of red pepper paste was determined using Trolox equivalent antioxidant capacity (TEAC) assay. A sample of 2 g was mixed with 20 mL of extraction solvent (70-mL methanol (99.9%, Merck KGaA, Germany) + 2-mL formic acid (99–100%, VWR, Germany) + 28-mL H_2_O) and stirred in a beaker with magnetic stirrer for 2 h. The mixture was centrifuged (Universal 320 R, Hettich Zentrifugen, Germany) at 21,380 × g for 15 min and the supernatant was decanted. About 10 μL of supernatant and 1 mL of ABTS^•+^ (2,2′-azino-bis(3-ethylbenzothiazoline-6-sulfonic acid)) (≥98%, HPLC grade, Sigma Aldrich, Germany) was mixed into the cuvette and absorbance was read at 734 nm with spectrophotometer (SPECORD 40, Analytik Jena AG, Germany). Trolox ((±)-6-hydroxy-2,5,7,8-tetramethylchroman-2-carboxylic acid) and *L*(+)-ascorbic acid (AppliChem, Germany) (Sigma Aldrich, Germany) was used as a standard and quality control, respectively. Results were expressed as μmol Trolox equivalent per g (μmol TE/g) of sample.

#### 2.6.4 Determination of color

Color of the samples was estimated using spectrophotometer (CM-600d, Konica Minolta, Japan) as described by Li et al. (2016). Sample of 6 g was evenly spread on a cuvette and color was analyzed at different points (n = 10) as reflected in CIELab (*L*∗, *a*∗, *b*∗) color space. All measurements were carried out to the CIE using the standard illuminant D65 and 10° observer at 20 ± 2 °C. Total color difference, *△E*∗, was estimated using Eq. (3):

(3) $$\Delta E^{*}=\sqrt{{\left(L^{*}-L_{0}^{*}\right)}^{2}+{\left(a^{*}-a_{0}^{*}\right)}^{2}+{\left(b^{*}-b_{0}^{*}\right)}^{2}}$$

where; *L∗* is lc *L∗*_0_ is lightness of untreated sample; *a∗* is redness/greenness of treated sample; *a∗*_0_ is redness/greenness of untreated sample; *b∗* is yellowness/blueness of treated sample; and *b∗*_0_ is yellowness/blueness of untreated sample.

### 2.7 Statistical data analysis

The relationship between independent variables and dependent variables was analyzed by using response surface regression procedure of Design-Expert software, version 10 (Stat-Ease Inc., MN, USA). Analysis of Variance (ANOVA) with F-test was performed to obtain the coefficients of the regression equation. Models developed included all the variables of polynomial regression at a significance level of *p* < 0.05. Response surface plots were drawn from the equations to further visualize the relationships of variables. Numerical optimization was implemented to find a good set of acceptable conditions. Measurements were done at least in triplicates with two technical replicates and results were reported as mean ± standard deviation (SD).

## 3 Results and discussions

### 3.1 Effect of HPP on microbiological quality of red pepper paste

As presented in Table 2, the highest reduction of AMB by 4.5 log CFU/g from the initial count of 6.6 log CFU/g was observed at pressure level of 527 MPa and holding time for 517 s. The yeasts and molds counts were reduced by 4.0 and 3.8 log CFU/g (detection limit of 1 log CFU/g) from the initial count of 5.0 and 4.8 log CFU/g for samples treated at 600 MPa for 315 s, respectively (Table 2). Yeasts and molds were comparatively more sensitive to HPP treatment than AMB. Similarly, Yuan et al. (2018) reported significant reduction of yeasts and molds below 1 log CFU/g and aerobic bacteria to <2 log CFU/g upon HPP treatment of aronia berry purée at 400 and 600 MPa for 5 min. Zhang et al. (2011) also reported that pressurizing yellow peaches at 600 MPa for 5 min reduced yeasts and molds below the detection limit.

**Table 2:** Effect of HPP on the microbiological quality of red pepper paste.

Run	Pressure [MPa]	Holding time [s]	Log reduction [log (N_o_/N)]		
			AMB	Yeasts	Molds
1	174	114	0.4 ± 0.0	0.9 ± 0.6	0.1 ± 0.5
2	527	517	4.5 ± 0.0	3.3 ± 0.5	3.5 ± 0.4
3	350	30	0.6 ± 0.1	1.0 ± 0.1	0.9 ± 0.5
4	350	600	1.9 ± 0.3	2.4 ± 0.1	2.2 ± 0.5
5	350	315	3.0 ± 0.1	1.9 ± 0.2	2.6 ± 0.2
6	174	517	0.4 ± 0.2	0.9 ± 0.0	0.1 ± 0.5
7	600	315	3.1 ± 0.6	4.0 ± 0.0	3.8 ± 0.0
8	100	315	0.2 ± 0.1	0.1 ± 0.4	0.6 ± 0.2
9	350	315	3.0 ± 0.1	1.9 ± 0.2	2.6 ± 0.2
10	527	114	0.7 ± 0.0	2.4 ± 0.2	2.7 ± 0.2

#### 3.1.1 Response surface modeling of AMB inactivation with HPP

Experimental results were analyzed in order to determine the effect of different levels of pressure and holding time on AMB, and fitted with the following quadratic Eq. (4):

(4) $$TPC=365-0.76A-1.11B-0.95AB+0.88A^{2}+0.57B^{2}$$

where; A is holding time, B is treatment pressure.

ANOVA results of the fitted quadratic model for AMB are presented in Table 3. A, B, AB, A^2^ and B^2^ were significant model terms at *p* < 0.05. The model F-value of 57.43 implied the model was significant with high correlation coefficient (R^2^ = 0.9863). This implied that 98.63% of the data variation was explained by the model. Le Man et al. (2010) suggested that R^2^ value should not be below 0.75 for a model to be adequate. Koocheki et al. (2009), however, argued that a high value of R^2^ does not necessarily indicate a good regression model and that such assumption could be taken on the basis of higher adjusted R^2^ value. According to Meyers et al. (2006), the difference in values of adjusted R^2^ and predicted R^2^ shall be less than 0.20.

**Table 3:** ANOVA for response surface quadratic model for AMB.

Source	Sum of squares	Df	Mean square	F-value	*p*-value
Model	21.88416	5	4.376831	57.43034	0.000814^a^
*A-Holding time*	*4.584334*	*1*	*4.584334*	*60.15308*	*0.001489*^a^
*B-Pressure*	*9.950013*	*1*	*9.950013*	*130.5585*	*0.000335*^a^
*AB*	*3.613912*	*1*	*3.613912*	*47.41974*	*0.002331*^a^
*A*^*2*^	*3.50488*	*1*	*3.50488*	*45.98908*	*0.002468*^a^
*B*^*2*^	*1.516723*	*1*	*1.516723*	*19.9016*	*0.011151*^a^
Residual	0.304845	4	0.076211		
*Lack-of-fit*	*0.299845*	*3*	*0.099948*	*19.98963*	*0.162616*^b^
*Pure error*	*0.005*	*1*	*0.005*		
Corrected total	22.189	9			

a Significant at *p* < 0.05.

b Not significant at *p* < 0.05.

In this study, the predicted R^2^ of 0.9031 was in reasonable agreement with the adjusted R^2^ of 0.9691. The high value of the adjusted R^2^ demonstrated a highly significant model implying the predicted and experimental values were in good agreement. Therefore, the model adequately predicted the observed data in the domain of the tested variables. The lack-of-fit F-value of 19.99 implied that it was not significant relative to the pure error. There was only a 16.26% chance that a “Lack of Fit F-value” this large could occur due to noise. Adequate precision ratios greater than 4 indicate adequate model discrimination (Tripathi and Mishra, 2011). Thus, the ratio of 19.647 demonstrated an adequate signal suggesting that the model could be used to navigate the design space.

**Figure 1:**
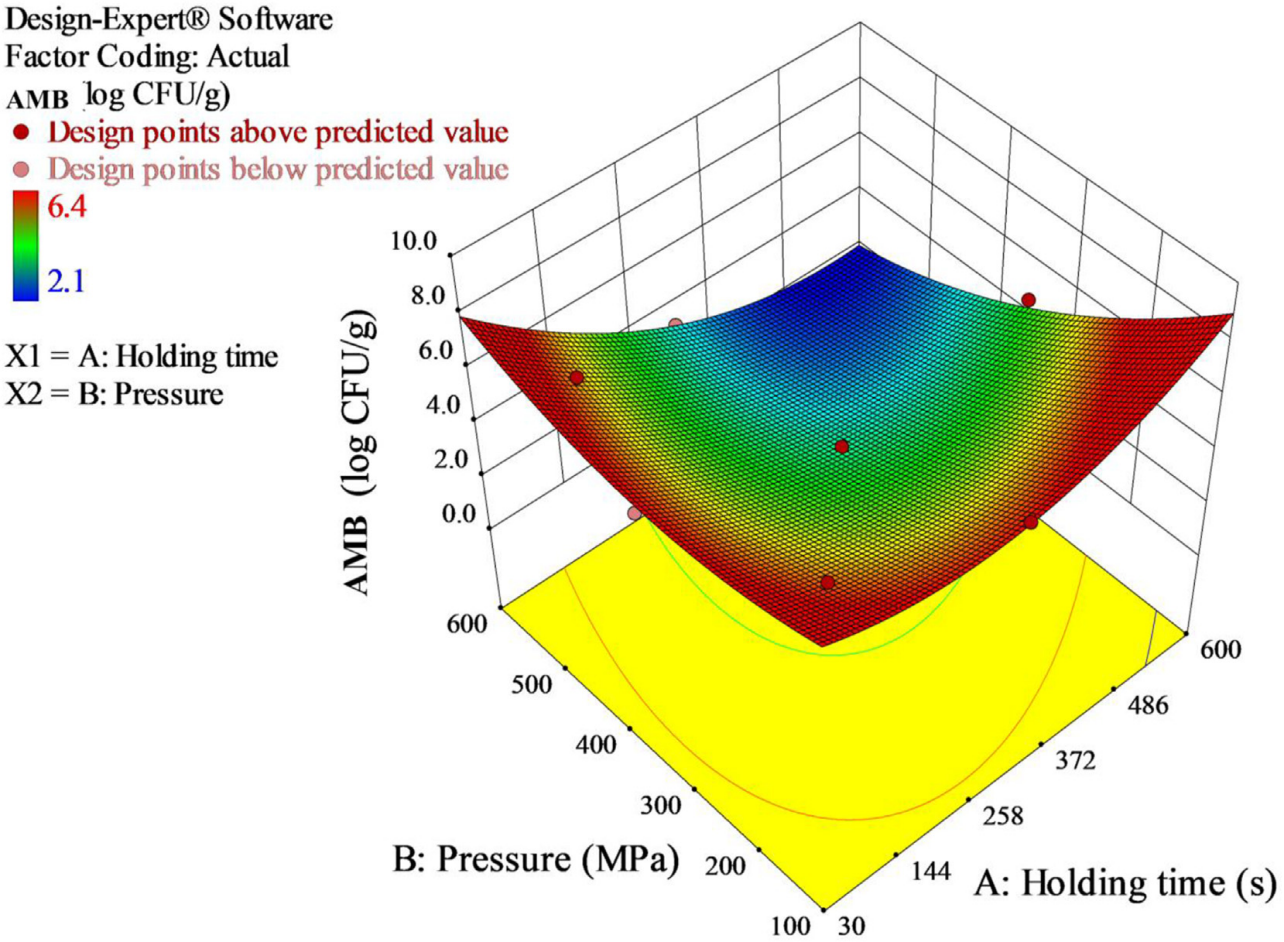
Response surface 3D plot of AMB inactivation as a function of pressure and holding time.

Three-dimensional response surface was plotted to evaluate the interaction between the variables for maximum reduction of AMB (Figure 1). As the pressure and holding time increased, the inactivation of AMB increased. Interaction effect between pressure and holding time had significantly (*p* < 0.05) reduced the AMB in red pepper paste. This indicated the dependence of microbial inactivation on pressure and holding time with curvilinear relationship between the parameters. However, operating at low holding time and high pressure as reported in several previous studies could be favorable for AMB inactivation.

#### 3.1.2 Response surface modeling of yeasts and molds with HPP

The variables and responses were analyzed to develop a suitable model. Quadratic and linear model equations, Eqs. (5) and (6) were established for yeasts and molds counts, respectively, at various levels of the pressure and holding time:

(5) $$Yeasts=3.44-0.41A-1.18B-0.27AB-0.042A^{2}-0.24B^{2}$$

(6) Molds=3.00−0.32A−1.33B

**Figure 2:**
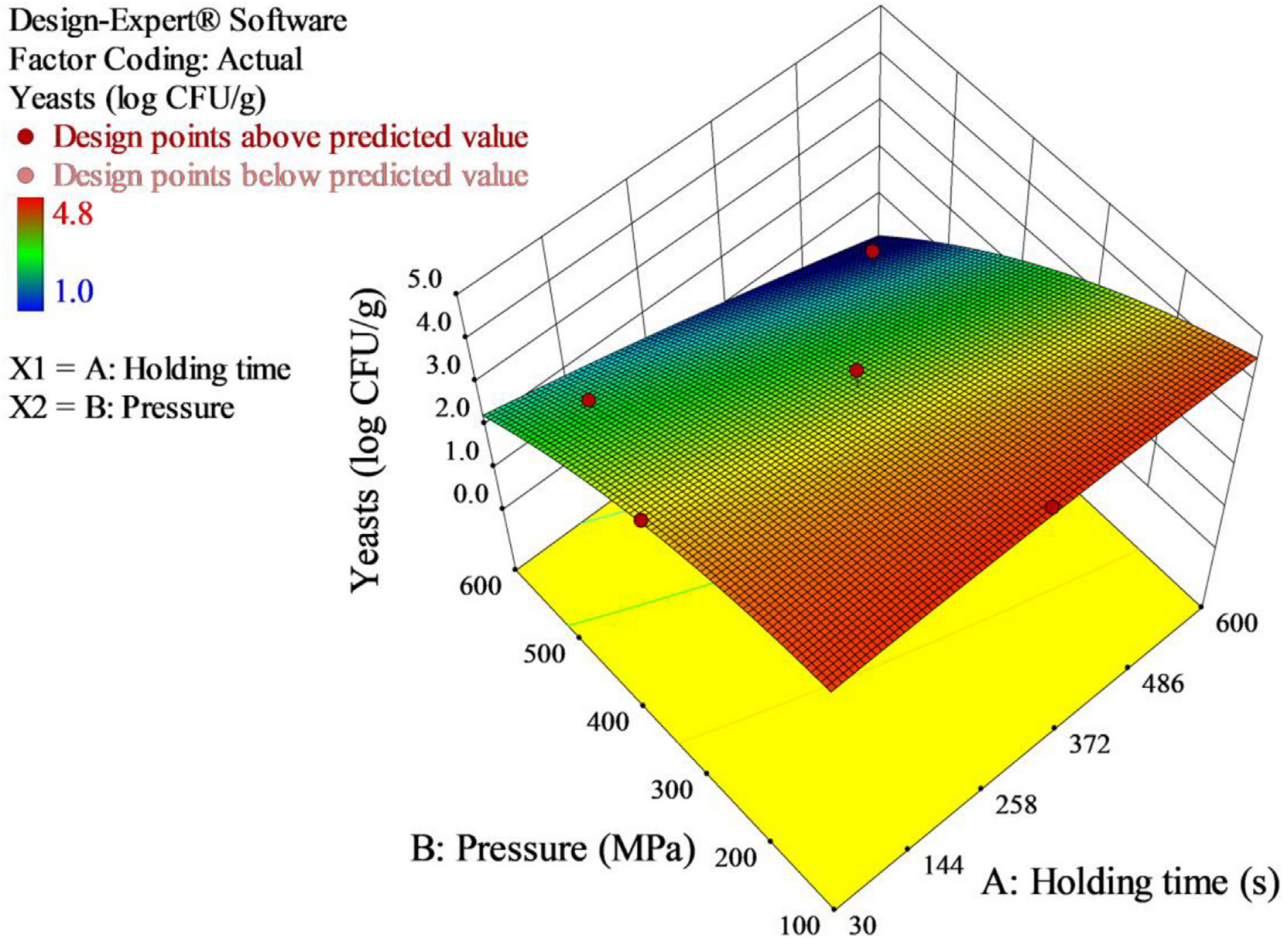
Response surface 3D plot of yeasts inactivation as a function of pressure and holding time.

Based on ANOVA of the developed model for yeast and mold counts, the models were significant as a result of F-values of 19.50 and 21.84, respectively. A and B model terms were significant for yeasts and only the B term was significant for molds, where other terms are non-significant. In Figure 2, it can be seen that pressure had a more pronounced impact than that of holding time. The “Lack of Fit F-value” of 0.40 having *p*-value of 0.7876 for yeasts indicated the lack-of-fit was not significant. There was a 78.76% chance that a “Lack of Fit F-value” this large could occur due to noise. The models for yeasts had higher R^2^ value (0.9606) than molds (0.8619). In the case of yeasts, the predicted R^2^ of 0.7754 was in reasonable agreement with the adjusted R^2^ of 0.9113; i.e. the difference is <0.20. Adequate precision ratio of 11.773 implied an adequate signal and the model could be used to navigate the design space.

Similarly, the adjusted R^2^ of 0.8224 was in reasonable agreement with the predicted R^2^ of 0.7567 for molds. Adequate precision ratio of 11.745 implied an adequate signal. The “Lack of Fit F-value” of 0.14 having *p*-value of 0.9610 indicated the lack-of-fit was not significant for molds. There is a 96.10% chance that a “Lack of Fit F-value” this large could occur due to noise. In both cases, however, pressure had significant impact in controlling the yeasts and molds count in red pepper paste samples.

### 3.2 Effect of HPP on the physicochemical properties of red pepper paste

#### 3.2.1 Effect of HPP treatment on total phenols

The effect of HPP on the physicochemical characteristics of red pepper paste samples is presented in Table 4. The total phenols (TP) content of the red pepper paste ranged from 0.28 to 0.33 g GAE/100 g. An increasing trend was observed among TP content at higher levels of pressure with longer holding times. An increase of 6.7% was obtained for both treatments at 527 MPa/517 s and 600 MPa/315 s when compared with the untreated sample.

**Table 4:** Effect of HPP on the physicochemical qualities of red pepper paste.

Run	Pressure [MPa]	Holding time [s]	Total phenols [g GAE/100 g]	Carotenoids [mg βc/100 g]	Antioxidants activity [μmol TE/g]	Color values			
						L∗	a∗	b∗	ΔE∗
Untreated	–	–	0.29 ± 0.01	96.0 ± 0.3	8.70 ± 0.02	29.99 ± 0.05	12.84 ± 0.06	8.90 ± 0.03	–
1	174	114	0.28 ± 0.03	96.5 ± 0.2	8.71 ± 0.13	30.01 ± 0.05	12.91 ± 0.11	7.85 ± 0.08	2.1
2	527	517	0.33 ± 0.01	98.4 ± 0.3	8.78 ± 0.05	31.82 ± 0.10	13.46 ± 0.10	7.04 ± 0.07	2.8
3	350	30	0.28 ± 0.03	97.2 ± 0.7	8.81 ± 0.08	30.03 ± 0.07	13.67 ± 0.12	8.89 ± 0.10	0.2
4	350	600	0.32 ± 0.02	97.9 ± 0.9	8.73 ± 0.15	31.94 ± 0.11	12.84 ± 0.07	5.88 ± 0.07	2.6
5	350	315	0.31 ± 0.02	97.3 ± 0.6	8.85 ± 0.06	30.94 ± 0.76	13.12 ± 0.06	7.55 ± 0.18	2.3
6	174	517	0.32 ± 0.02	97.7 ± 0.8	8.83 ± 0.05	31.30 ± 0.11	12.94 ± 0.12	6.35 ± 0.11	2.4
7	600	315	0.33 ± 0.01	98.3 ± 0.7	8.91 ± 0.02	31.83 ± 0.10	13.75 ± 0.16	7.50 ± 0.08	1.8
8	100	315	0.28 ± 0.02	96.7 ± 0.1	8.75 ± 0.13	29.89 ± 0.09	12.62 ± 0.04	7.51 ± 0.09	2.6
9	350	315	0.29 ± 0.03	97.1 ± 0.4	8.78 ± 0.12	30.97 ± 0.05	13.22 ± 0.13	7.84 ± 0.13	2.5
10	527	114	0.32 ± 0.02	97.3 ± 0.5	8.95 ± 0.03	30.99 ± 0.08	14.12 ± 0.05	8.34 ± 0.06	0.6

Several works have reported an increase in the TP content of various food products after HPP treatment. Varela-Santos et al. (2012) reported that HPP treatment of fresh pomegranate juice from 350 to 550 MPa for 30–150 s significantly increased the TP content in the range of 3.38–11.99%. Increase in 12% TP content was reported for onion paste treated at 400 MPa at 5 °C for 5 min (Roldán-Marín et al., 2009). The increase in the concentration of TP could be due to the improved extractability of some phenolic compounds as a function of instantaneous pressure drop (Varela-Santos et al., 2012; Gómez-Maqueo et al., 2020). Additionally, the increase in TP in this study might be explained by the disruption of hydrophobic bonds with cell membrane and cell wall which might lead to enhanced cell permeability and mass transfer, releasing matrix-bound phenolics, as also documented in other investigations (Patras et al., 2009a; Wang et al., 2012).

The TP content for the entire domain was modeled through a linear regression as shown with Eq. (7).

(7) TP=0.30+0.011A+0.018B

Both A and B are highly significant model terms, indicating holding time and pressure contributed to the change in TP content. The model was highly significant in contrast with the lack-of-fit. The data fitted well with the established model with R^2^ of 0.8653. The adjusted R^2^ of 0.8268 was in reasonable agreement with the predicted R^2^ of 0.7755. Adequate precision ratio of 12.019 implied an adequate signal.

#### 3.2.2 Effect of HPP treatment on carotenoids

Carotenoids content of the red pepper paste ranged from 96.0 to 98.4 mg βc/100 g (Table 4). A slight and non-significant increase in carotenoids (2.5%) was observed with increased pressure and holding time. Related study by De Ancos et al. (2000) reported that HPP treatments of rojo brillante purée at 50 and 300 MPa for 15 min at 25 °C and of sharon purée at 50 and 400 MPa for the same time and at the same temperature resulted in a significant increase in the carotenoid content in the range of 9%–27%. In contrast, non-significant changes in the total carotenoids or β-carotene were reported for HPP-treated food products such as carrot and broccoli (McInerney et al., 2007), papaya beverage (Chen et al., 2015), pumpkin purée (González-Cebrino et al., 2016) and mango pulp (Liu et al., 2016). Depending on the respective matrix, this could be due to the limited effect of HPP on covalent bonds in low molecular mass compounds like carotenoids.

The carotenoids content for the entire domain was modeled through a linear regression as shown with Eq. (8).

(8) Carotenoids=97.44+0.41A+0.47B

As observed from ANOVA, the estimated *F*-value (23.76) indicates a highly significant (*p* < 0.0008) regression model. Both A and B model terms were highly significant, whereas the lack-of-fit was not statistically significant due to the lower *F*-value (3.67). The data were also in good fit with developed model having R^2^ of 0.8716. The adjusted R^2^ of 0.8349 is in reasonable agreement with the predicted R^2^ of 0.7385. Adequate precision ratio of 12.553 implies an adequate signal.

#### 3.2.3 Effect of HPP treatment on antioxidants activity

**Figure 3:**
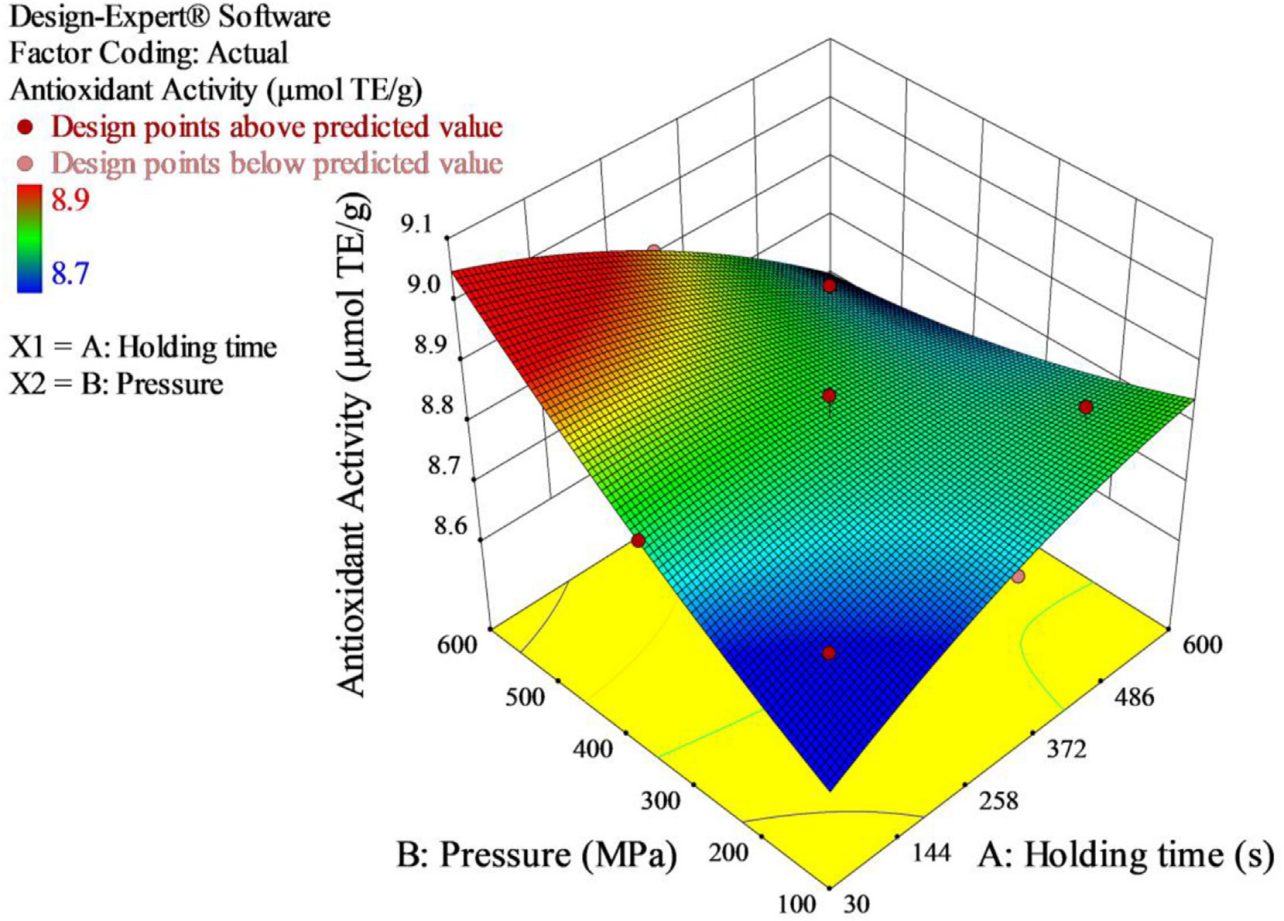
Response surface 3D plot of antioxidants activity as a function of pressure and holding time.

The antioxidants activity of the red pepper paste ranged from 8.70 to 8.95 μmol TE/g (Table 3). A slight increase of 2.9% in the antioxidants activity was observed with increased treatment pressure (Figure 3). The increase could be attributed to the release of compounds with antioxidant properties into the extracellular environment, as a result of cell wall disruption triggered by HPP treatment (Briones-Labarca et al., 2011). Similar findings have been reported already, demonstrating an increase in antioxidants activity of food products after HPP treatment, often accompanied by increased concentration of compounds having antioxidant properties. Patras et al. (2009a) and Patras et al. (2009b) reported that HPP treatments of blackberry, carrot, and tomato purées from 400 to 600 MPa at 10 °C–30 °C for 15 min significantly increased the antioxidants activity by 29–68%, 22–37% and 8–27% in comparison to untreated samples, respectively. In a different study, García-Parra et al. (2011) evaluated HPP treatment of nectarine purée at 450 and 600 MPa at 10 °C for 5 and 10 min and observed significantly higher values for antioxidants activity when compared to untreated sample. Following HPP treatment of cashew juice at 250 MPa for 3 min, Queiroz et al. (2010) also observed an increase in antioxidants activity by 40%.

The antioxidants activity of red pepper paste was modeled with a quadratic regression as shown with Eq. (9).

(9) $$Antioxidant\;activity=8.82-0.021A+0.052B-0.072\text{AB}-0.018A^{2}+0.012B^{2}$$

Based on ANOVA, the model was significant as a result of F-value of 1065. The factors that had significant effects (*p* < 0.05) on antioxidants activity were the linear term (B) and the interaction term (AB). The linear term (A) and the quadratic terms (A^2^ and B^2^) did not make any significant contribution towards antioxidants activity. The “Lack of Fit F-value” of 0.17 with *p*-value of 0.9036 indicated non-significant lack-of-fit. There was a 90.36% chance that a “Lack of Fit F-value” this large could occur due to noise. The higher value of R^2^ (0.9301) suggests a good fit and that only 7% of variation for the response could not be explained by the model. Adjusted R^2^ (0.8427) was in reasonable agreement with the predicted R^2^ (0.6458). Adequate precision ratio of 10.518 implied an adequate signal and the model could be used to navigate the design space.

#### 3.2.4 Effect of HPP treatment on color

**Figure 4:**
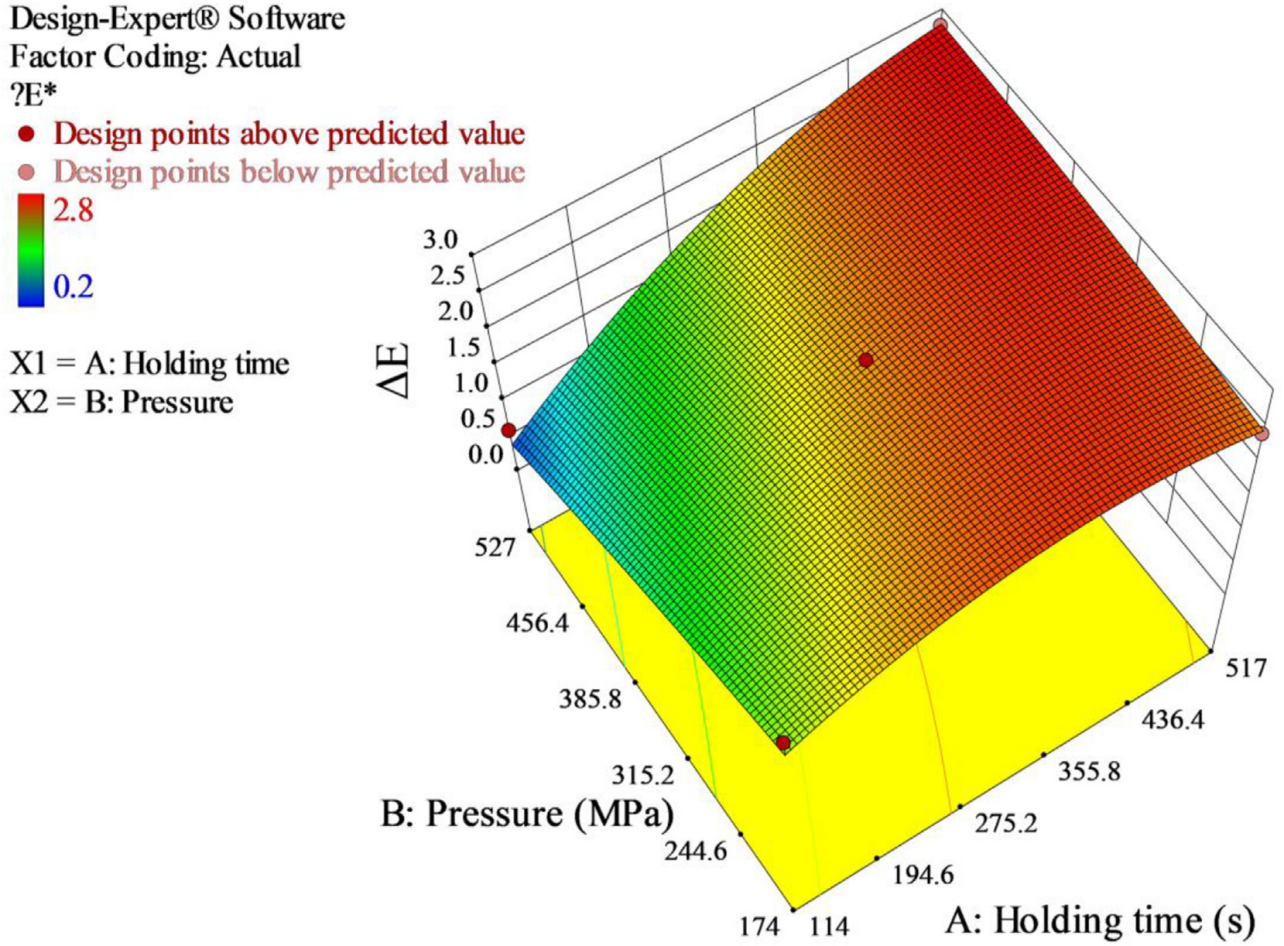
Response surface 3D plot of *ΔE∗* as a function of pressure and holding time.

**Figure 5:**
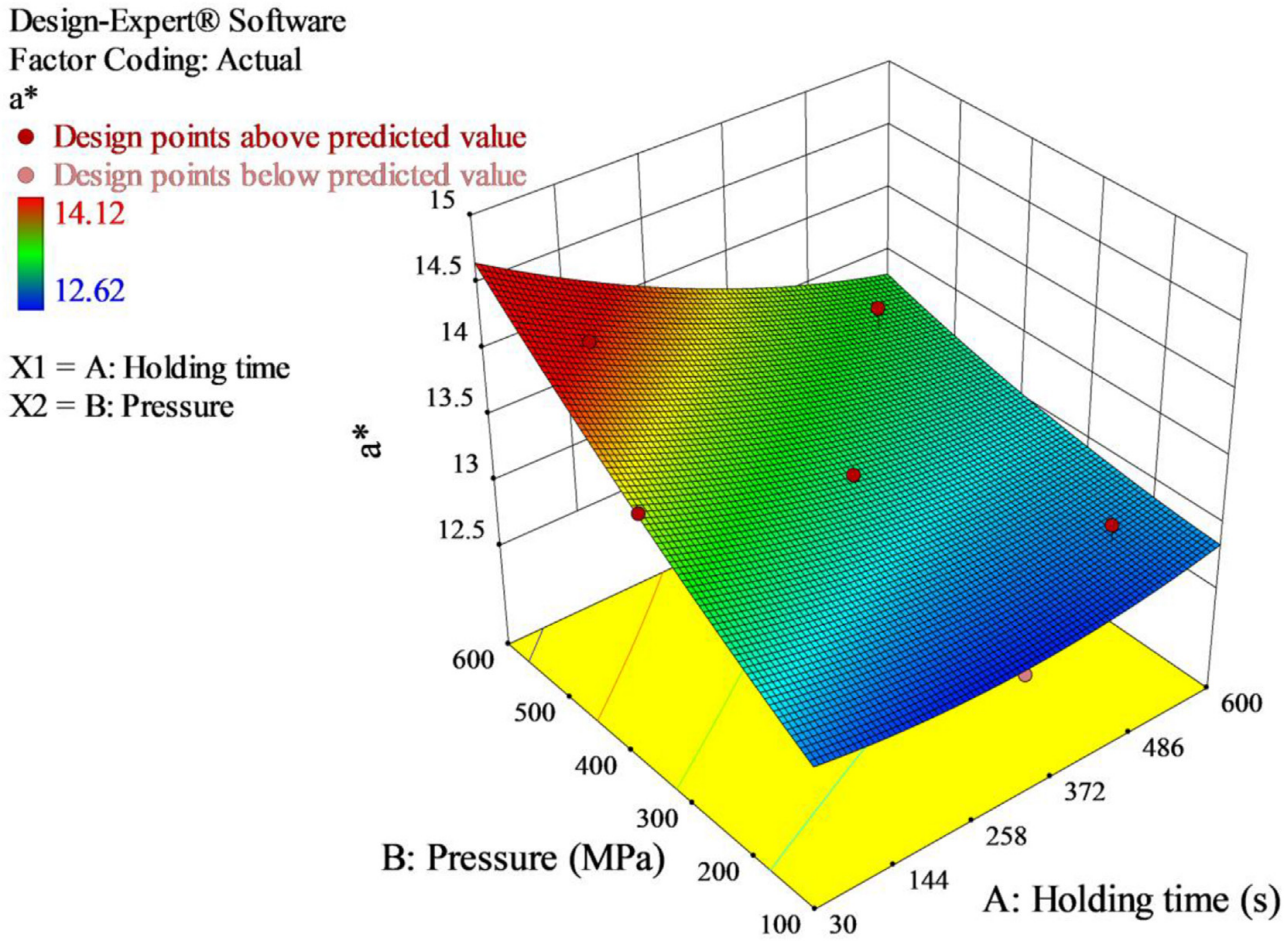
Response surface 3D plot of *a∗* as a function of pressure and holding time.

The CIELab values of the untreated red pepper paste were *L∗* = 29.99, *a∗* = 13.84, and *b∗* = 8.90 (Table 4). At all the combinations of pressure-holding time, the *L∗*, *a∗* and *b∗* values ranged from 29.89 to 31.83, 12.62 to 14.12 and 5.88 to 8.89, respectively. The *ΔE∗* value ranged from 0.2 to 2.8. According to Cserhalmi et al. (2006), *ΔE∗* values could be categorized as ‘imperceptible’, ‘slightly noticeable’, ‘noticeable’, ‘well visible’ and ‘great’ to the corresponding lower limit of 0.0, 0.5, 1.5, 3.0 and 6.0, respectively. Thus, *ΔE∗* values in this study were within the range of “imperceptible” to ‘noticeable’. With increasing pressure and holding time, the values of *ΔE∗* increased (Figure 4), the changes in color of red pepper paste become more visible. The observed differences can be related to a slight increase in lightness (*L∗*) and (*a∗*) values describing red/green color observed at higher pressure and longer holding time treatments. In the 3D response surface plot, it is also illustrated that holding time had lower contribution for the change of *a∗* values relative to pressure (Figure 5).

Findings of the study are in line with several reports on fruit products. For instance, litchi arils (Phunchaisri and Apichartsrangkoon, 2005), tomato purée (Sánchez-Moreno et al., 2006), Granny Smith apple purée (Landl et al., 2010), and mango pulp (Kaushik et al., 2014), have already been reported to show increased *L∗* values with increasing pressure. The *a∗* values of HPP-treated tomato and carrot purées from 400 to 600 MPa at 10–30 °C for 15 min were higher than untreated samples (Patras et al., 2009a, 2009b). Similarly, Unni and Chauhan (2018) observed increased *a∗* values of onion paste when treated from 200 to 600 MPa. In another study by Unni et al. (2014), garlic paste treated from 200 to 600 MPa demonstrated a significant decrease of *b∗* values. The higher values of *a∗* in this study suggested that the red pepper paste was rich in yellow-red-colored components, which is in agreement with the higher amount of carotenoids and phenols, as reported earlier in section 3.2.

ANOVA of the color values revealed that the linear model Eq. (10) for *L∗* is highly significant (*p* < 0.0001).

(10) $$L^{*}=30.97+0.60A+0.53B$$

The A and B model terms were also highly significant indicating holding time and pressure contributing to significant changes of *L∗* values. The “Lack of Fit F-value” of 120.53 with *p*-value of 0.0696 indicated non-significant lack-of-fit compared to the pure error. There was a 6.96% chance that a “Lack of Fit F-value” this large could occur due to noise. The model had higher R^2^ value of 0.9406 suggesting a good fit of the model. Adjusted R^2^ value of 0.9236 was in reasonable agreement with predicted R^2^ value of 0.8594. Adequate precision ratio of 19.172 indicated an adequate signal and the model can be used to navigate the design space.

ANOVA of the color values indicated that the quadratic model Eq. (11) for *a∗* value was highly significant (*p* < 0.0069).

(11) $$a^{*}=13.17-0.23A+0.42B-0.17AB+0.077A^{2}+0.042B^{2}$$

The linear model terms (A and B) were significant indicating holding time and pressure made significant changes towards *a∗* values. In contrast, the interaction (AB) and quadratic model terms (A^2^ and B^2^) were not significant. The “Lack of Fit F-value” of 5.12 with *p*-value of 0.3115 indicated non-significant lack-of-fit. There was a 31.15% chance that a “Lack of Fit F-value” this large could occur due to noise. The model had higher R^2^ value of 0.9595 suggesting good fit of the model. Adjusted R^2^ value of 0.9088 was in reasonable agreement with predicted R^2^ value of 0.7197. Adequate precision ratio of 12.979 implied an adequate signal and the model can be used to navigate the design space.

ANOVA of the color values revealed that the linear model Eq. (12) for *b∗* was highly significant (*p* < 0.0002).

(12) $$b^{*}=7.47-0.88A+0.15B$$

The linear model term (A) was significant indicating holding time made significant changes towards *b∗* values; while the B term is not significant. The “Lack of Fit F-value” of 2.35 with *p*-value of 0.4616 indicated non-significant lack-of-fit. There was a 46.16% chance that a “Lack of Fit F-value” this large could occur due to noise. The model had higher R^2^ value of 0.9097 suggesting good fit of the model. Adjusted R^2^ of 0.8839 was in reasonable agreement with predicted R^2^ of 0.8086. Adequate precision ratio of 15.127 implied an adequate signal and the model can be used to navigate the design space.

ANOVA of the color values revealed that the quadratic model Eq. (13) for *ΔE∗* was highly significant (*p* < 0.0027) (Table 5).

(13) $$\Delta E^{*}=2.40+0.758A-0.27B+0.46AB-0.46A^{2}-0.061B^{2}$$

**Table 5:** ANOVA for response surface linear model for *ΔE∗* color values.

Source	Sum of squares	Df	Mean square	F-value	*p*-value
Model	7.08	5	1.42	31.25	0.0027^a^
*A-Holding time*	*4.56*	*1*	*4.56*	*100.66*	*0.0006*^a^
*B-Pressure*	*0.59*	*1*	*0.59*	*13.08*	*0.0224*^a^
*AB*	*0.85*	*1*	*0.85*	*18.86*	*0.0122*^a^
*A*^*2*^	*0.96*	*1*	*0.96*	*21.19*	*0.0100*^a^
*B*^*2*^	*0.017*	*1*	*0.017*	*0.37*	*0.5750*^a^
Residual	0.18	4	0.045		
*Lack-of-fit*	*0.16*	*3*	*0.054*	*2.69*	*0.4151*^b^
*Pure error*	*0.020*	*1*	*0.020*		
Corrected total	7.26	9			

a Significant at *p* < 0.05.

b Not significant at *p* < 0.05.

The linear (A and B), the interaction (AB) and the quadratic (A^2^) model terms were significant indicating pressure and holding time made significant changes towards *ΔE∗* values. Only the quadratic model term (B^2^) of pressure is not significant. The “Lack of Fit F-value” of 2.69 with *p*-value of 0.4151 indicated non-significant lack-of-fit compared to the pure error. There was a 41.51% chance that a “Lack of Fit F-value” this large could occur due to noise. The model had high R^2^ value of 0.9750 suggesting good fit of the model. Adjusted R^2^ of 0.9438 was in reasonable agreement with predicted R^2^ of 0.8312. Adequate precision ratio of 14.765 implied an adequate signal and the model can be used to navigate the design space.

### 3.3 Process optimization and model validation

The criteria chosen to optimize process and response variables were based on desirable responses as presented in Table 6.

**Table 6:** Criteria set for optimization of process and response variables for HPP treatment of red pepper paste.

Process and response variables	Goal	Lower range	Upper range
Holding time (s)	In range	30	600
Pressure (MPa)	In range	100	600
AMB (CFU/g)	Minimize	2.1	6.4
Yeasts (CFU/g)	Minimize	1.0	4.8
Molds (CFU/g)	Minimize	1.0	4.7
TP (g GAE/100 g)	Maximize	0.28	0.33
Carotenoids (mg βc/100 g)	Maximize	96.5	98.4
Antioxidants activity (μmol TE/g)	Maximize	8.71	8.95
ΔE∗	Minimize	0.2	2.8

The criteria for numerical solution were analyzed by running the optimum solution with the highest desirability for validation of the model and average values of responses compared with predicted ones are presented in Table 7. The percentage of relative errors varied from 0.7 to 16.7%. Although the highest percentage of relative error was for $\Delta E^{*}$, both the predicted and experimental values are within the range of ‘slightly noticeable’, i.e. 0.5–1.5. Thus, the results verify that the experimental data were comparable with the predicted data. Moreover, optimization of HPP conditions suggest that the maximum desirability (0.622) that meets all the goals presented in Table 6 can be achieved through treatment of the red pepper paste at 536 MPa for 125 s.

**Table 7:** Model validation with optimum HPP treatment conditions on the microbiological and physicochemical qualities of red pepper paste.

	Holding time [s]	Pressure [MPa]	Microbial load, Log [CFU/g]			Total phenols [g GAE/100 g]	Carotenoids [mg βc/100 g]	Antioxidants activity [μmol TE/g]	*ΔE∗*
			AMB	Yeasts	Molds				
Predicted	125	536	3.3	2.5	2.1	0.34	97.7	8.95	0.6
Experimental	125	536	3.4 ± 0.2	2.3 ± 0.1	2.3 ± 0.3	0.33 ± 0.01	96.6 ± 0.2	9.01 ± 0.02	0.7
Relative error (%)			3.0	8.0	9.5	2.9	1.1	0.7	16.7

## 4 Conclusions

Pressure and holding time significantly reduced the AMB, yeasts and molds counts following HPP treatment. The technique was also found to enhance retention of phytochemicals such as total phenols, carotenoids and antioxidants activity. Whereas, ‘noticeable’ changes for *ΔE∗* values was observed within the domains of pressure and holding time. The models developed as function of pressure and holding time could be used to successfully predict the quality characteristics of red pepper paste. The present approach offered an optimum solution for red pepper paste treatment at 536 MPa for 125 s at desirability value of 0.622 with reduced number of experiments. The validation data successfully verified the adequacy of the model for prediction of optimum response values. Owing to its efficiency, HPP could be used as a preservation technique to enhance the microbiological safety of red pepper paste and develop healthier food products for consumers.

## Declarations

### Author contribution statement

Henock Woldemichael Woldemariam: Conceived and designed the experiments; Performed the experiments; Analyzed and interpreted the data; Contributed reagents, materials, analysis tools or data; Wrote the paper.

Shimelis Admassu Emire, Paulos Getachew Teshome: Conceived and designed the experiments; Wrote the paper.

Stefan Toepfl: Conceived and designed the experiments; Contributed reagents, materials, analysis tools or data.

Kemal Aganovic: Conceived and designed the experiments; Analyzed and interpreted the data; Contributed reagents, materials, analysis tools or data; Wrote the paper.

### Funding statement

This research did not receive any specific grant from funding agencies in the public, commercial, or not-for-profit sectors.

### Data availability statement

Data will be made available on request.

### Declaration of interest’s statement

The authors declare no competing interests.

### Additional information

No additional information is available for this paper.
